# Gene variants and clinical characteristics of children with sitosterolemia

**DOI:** 10.1186/s12944-024-02077-1

**Published:** 2024-03-20

**Authors:** Rui Gu, Hui Wang, Chun-Lin Wang, Mei Lu, Miao Miao, Meng-Na Huang, Yi Chen, Yang-Li Dai, Ming-Qiang Zhu, Qiong Zhou, Chao-Chun Zou

**Affiliations:** 1 Department of Endocrinology, Children’s Hospital of Zhejiang University School of Medicine, National Clinical Research Center for Child Health, Zhejiang, China; 2 Department of NICU, Sir Run Run Shaw Hospital, Zhejiang University School of Medicine, Zhejiang, China; 3 Department of Rehabilitation, Children’s Hospital of Zhejiang University School of Medicine, Zhejiang, China; 4 Department of Pediatrics, The First Affiliated Hospital, Zhejiang University School of Medicine, Zhejiang, China; 5 Department of Pediatrics, Women and Children’s Hospital, School of Medicine, Xiamen University, Xiamen, China; 6 Department of Pulmonology, Children’s Hospital of Zhejiang University School of Medicine, Zhejiang, Hangzhou China; 7 Department of Pediatrics, Hangzhou Children’s Hospital, Hangzhou, 310005 China

**Keywords:** Sitosterolemia, Genotype, Phenotype, Diagnosis, Treatment

## Abstract

**Objective:**

To enhance the detection, management and monitoring of Chinese children afflicted with sitosterolemia by examining the physical characteristics and genetic makeup of pediatric patients.

**Methods:**

In this group, 26 children were diagnosed with sitosterolemia, 24 of whom underwent genetic analysis. Patient family medical history, physical symptoms, tests for liver function, lipid levels, standard blood tests, phytosterol levels, cardiac/carotid artery ultrasounds, fundus examinations, and treatment were collected.

**Results:**

The majority (19, 73.1%) of the 26 patients exhibited xanthomas as the most prevalent manifestation. The second most common symptoms were joint pain (7, 26.9%) and stunted growth (4, 15.4%). Among the 24 (92.3%) patients whose genetics were analyzed, 16 (66.7%) harbored *ABCG5* variants (type 2 sitosterolemia), and nearly one-third (8, 33.3%) harbored *ABCG8* variants (type 1 sitosterolemia). Additionally, the most common pathogenic *ABCG*5 variant was c.1166G > A (p.Arg389His), which was found in 10 patients (66.7%). Further analysis did not indicate any significant differences in pathological traits among those carrying *ABCG*5 and *ABCG*8 variations (*P* > 0.05). Interestingly, there was a greater abundance of nonsense variations in *ABCG*5 than in *ABCG*8 (*P* = 0.09), and a greater frequency of splicing variations in *ABCG*8 than *ABCG*5 (*P* = 0.01). Following a change in diet or a combination of ezetimibe, the levels of cholesterol and low-density lipoprotein were markedly decreased compared to the levels reported before treatment.

**Conclusion:**

Sitosterolemia should be considered for individuals presenting with xanthomas and increased cholesterol levels. Phytosterol testing and genetic analysis are important for early detection. Managing one’s diet and taking ezetimibe can well control blood lipids.

## Introduction

Sitosterolemia is a rare genetic condition in which the body has higher than normal levels of phytosterols [1], specifically sitosterol, cholestanol, campesterol and stigmasterol. Elevated blood lipid levels, such as those of low-density lipoprotein cholesterol (LDL-C) and cholesterol, are typically associated with sitosterolemia. It affects only approximately 1 in a million people [2], and more than 100 cases have been reported worldwide [3], with many of these cases occurring in East Asian nations. Type 2 sitosterolemia (*ABCG*5) or type 1 sitosterolemia (*ABCG*8) gene variations are the cause of sitosterolemia [4], and the *ABCG*5 and *ABCG*8 transporter proteins are highly expressed in the human liver and small intestine [5]. The *ABCG*5 and *ABCG*8 genes are located at the sitosterolemia locus on chromosome 2p21. The *ABCG*5/8 heterodimer allows for the removal of phytosterols from cells in the small intestine, transporting them into the intestinal cavity [6]. Xenosterols accumulate as a result of deficiencies in the efflux pump, and patients with this condition have plasma levels of xenosterols that are up to 40 times greater than those of healthy people [7]. Elevated amounts of xenosterol are present in all sitosterolemia patients. Nevertheless, the increase in plasma cholesterol appears to be negatively correlated with age, but the underlying process is still unclear. According to certain research, a juvenile gut may absorb cholesterol at a greater rate than an adult intestine [8]. Sitosterolemia in children can cause mainly xanthomas [9], mildly increased cholesterol and LDL-C levels, premature atherosclerotic disease, macrothrombocytopenia, or anemia. There have also been reports of other rare signs, such as growth retardation and thyroid dysfunction, arthritis, liver disease (hepatosplenomegaly with aberrant hepatic function) and cardiac disease [10, 11].

In many parts of China, it is not possible to measure plasma phytosterol levels. This poses a challenge in accurately diagnosing sitosterolemia and ultimately prolongs the time it takes to do so. Both slightly elevated or normal cholesterol levels can make it easy to miss a diagnosis or mistakenly diagnose the condition as familial hypercholesterolemia (FH) [12]. Furthermore, research has shown that sitosterol alone can lead to cardiovascular damage [13–16], underscoring the critical need for early intervention. However, without an accurate diagnosis of sitosterolemia, controlling phytosterol levels may prove difficult, particularly in patients with normal cholesterol levels. To highlight the importance of identifying and treating sitosterolemia at an early stage, the clinical and genetic features of 26 patients with this rare condition were examined thoroughly. The clinical characteristics of patients with sitosterolemia with different common pathogenic variants were also evaluated to explore whether there were differences between the different variants.

## Materials & methods

### Research project

Between 2013 and 2023, the Children’s Hospital of Zhejiang University School of Medicine, the First Affiliated Hospital of Zhejiang University School of Medicine and Women and Children’s Hospital of Xiamen University made diagnoses of patients with sitosterolemia, a genetic or blood condition characterized by high levels of phytosterol. A striking 80.8% of these patients (21 individuals) were treated at the Children’s Hospital of Zhejiang University School of Medicine.

The Ethics Committee of the Children’s Hospital of Zhejiang University School of Medicine approved this study.

### Clinical information collection

Clinical information, such as demographic details (including sex and age), age of onset, clinical characteristics (such as xanthomas, fundus disease, abnormal morphology of the liver and spleen, arthralgia and stunted growth), and patient family medical history, was collected. Standard blood tests, tests for liver function, and measurements of the levels of lipids, such as TC, LDL-C, lipoprotein a (Lp(a)) or phytosterols (blood was drawn together and sent to Fudan University Affiliated Children’s Hospital for mass spectrometry monitoring), were also performed. The patients also underwent a range of diagnostic procedures, including ultrasound to gauge the thickness of the middle layer and identify the presence of plaque in the carotid arteries and cardiac arteries, along with thorough fundus examinations.

### Genetic analysis

Sequencing was completed on the Illumina sequencing platform. The obtained data included known gene exons and upstream and downstream 5 bp sequences in the human genome. The average sequencing depth was ≥90X, with approximately 98% of the target sequences having a sequencing depth greater than 20X. Base recognition was performed on all the sequencing segments. This test was conducted by establishing and validating domain medicine.

The secondary analysis was performed mainly using the GATK software suite for sequencing data analysis. The sequence was paired with the UCSC hg19 reference genome via BWA.

Single-nucleotide variation and long-segment insertion/deletion variation analysis were performed using VEP software (Variable Effect Predictor) to analyze the variation based on genetic disease databases, such as ClinVar, OMIM, HGMD, gnomAD, variant databases, and large-scale sequencing data. The library screens for variants and uses various recognized computer algorithms to predict and classify the potential pathogenicity of variants.

The American College of Medical Genetics and Genomics (ACMG) provided guidelines on how to interpret the data, including criteria for assessing the data. The HGVS suggests guidelines for naming the different alternatives.

### Statistical analysis

We utilized the SPSS version 23 and GraphPad 9 programs for conducting our statistical analyses. The data with the most variation and unpredictability are presented as the average ± the standard degree of deviation (SD) and were evaluated using the independent t test. For data that did not follow a normal distribution, we reported the middle value and the range of its distribution and employed the Mann–Whitney test for evaluation. The counting data are presented as proportions and were subjected to precise FISH analysis. We considered differences with a *P* value < 0.05 to be statistically significant.

## Results

### Demographics

Among the 26 individuals included in the study (as shown in Table 1), 13 were male, and 13 were female. The range of age at initiation varied widely between 5 days and 9.50 years, with a central age of 4.09 years. Among all patients, only 2 (7.7%) were younger than 1. Sixteen (61.5%) patients were aged between 1 and 6 years, and 8 (30.8%) patients were aged between 6 and 10 years. The diagnostic age ranged from 1 to 12 years, with the middlemost age being 5.92 years. There were no patients younger than 1 year. Among the 26 patients, 16 (61.5%) were between the ages of 1 and 6, and the remaining 10 (38.5%) were 6–12 years old. Seven individuals (26.9% of the total) had a genetic background of elevated lipid levels in the blood.

**Table 1:**
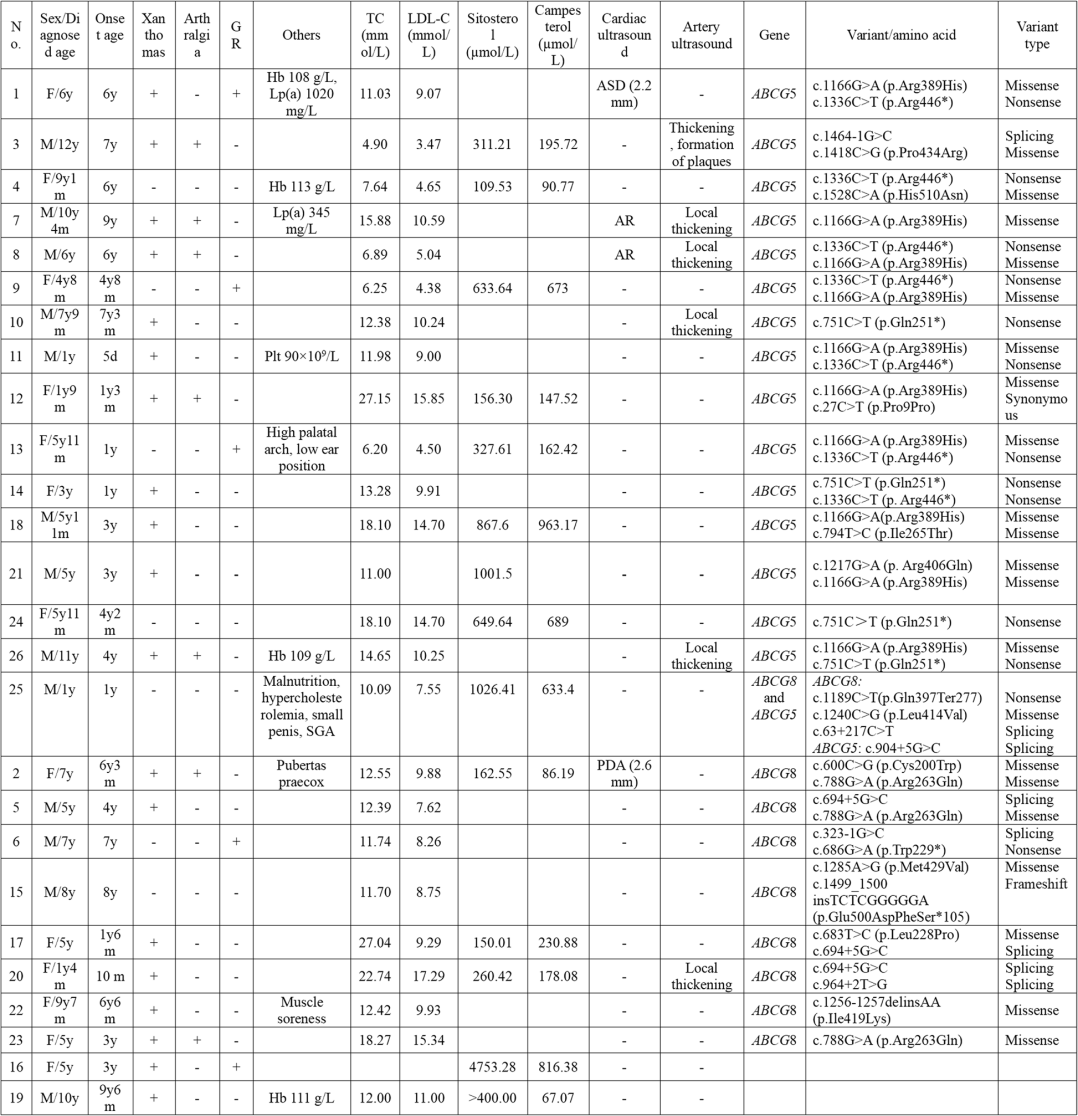
Summary of the clinical data of 26 patients

*M* Male, *F* Female, *Y* Year, *M* Month, *D* Day, *GR* Growth retardation, *Hb* Hemoglobin, *Plt* Platelet, *Lp(a)* Lipoprotein a, *ASD* Atrial septal defect, *AR* Aortic regurgitation, *PDA* Patent foramen ovale, *SGA* Small for gestational age infant

Patients 8 and 9 were brother and sister, respectively. The reference intervals for phytosterol levels were as follows: sitosterol (μmol/L): 0-2Y: 0.01–15.49; 3-6Y: 0.01–15.44; 7-15Y: 0.01–10.00; and campesterol (μmol/L): 0-2Y: 0.01–15.67; 3-6Y: 0.01–17.96; and 7-15Y: 0.01–10.39. All patients with one variant in our table were homozygous

### Clinical manifestations and laboratory examination

**Fig. 1:**
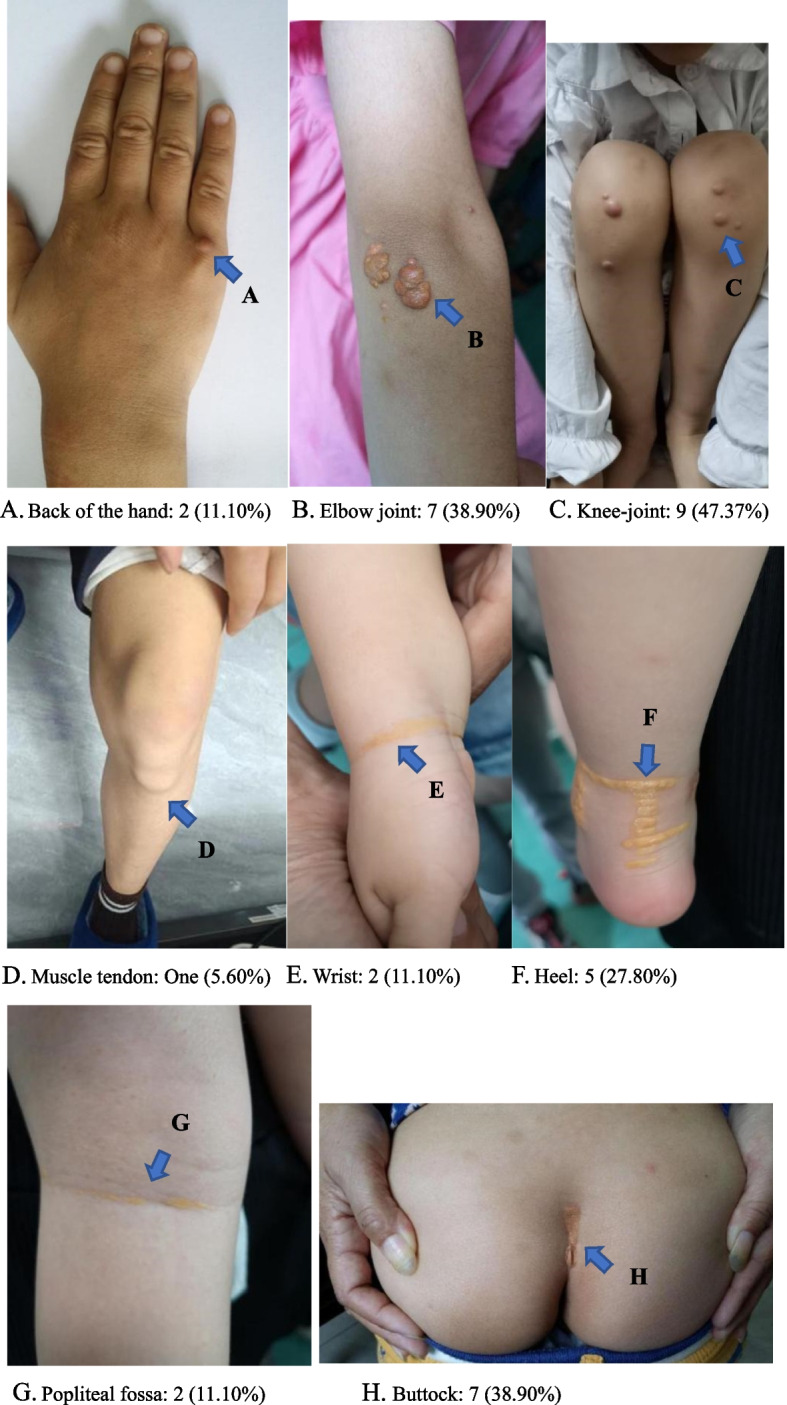
Images of xanthomas in different positions on the body of pediatric patients

Among the 23 individuals who showed symptoms, the majority (19, 82.6%) had xanthomas as the most prevalent manifestation. Among a total of 19 patients, 9 (47.4%) had xanthomas of the knee joint. There were 7 (38.9%) xanthomas in the elbow joint and buttock, 5 (27.8%) in the heel, and 4 (22.2%) each in the arm, leg and ankle. Less common locations included the popliteal fossa, back of the hand, and wrist, with 2 (11.1%) xanthomas each. Remarkably, one (5.6%) lesion was buried within the muscle tendon (Fig. 1A-H). Among the 19 individuals with xanthomas, 11 (57.9%) had fusion, and nearly half (9, 47.4%) had mysterious linear golden rashes. Xanthomas were observed in patients who were as young as 5 days and as mature as 9 years, with an average age of 3 years. Several other indications were observed, including joint pain in 7 patients (26.9%) and stunted growth in 4 patients (15.4%).

Among the 26 patients, 57.7% (15 patients) had previously undergone testing for sitosterol and campesterol in their serum either before beginning medication or after starting food control. The serum sitosterol levels varied greatly, ranging from a minimum of 156.30 to a maximum of 4753.28 μmol/L; the average value was high at 764.98 ± 1193.

The concentration of campesterol exhibited a wide range, with values fluctuating between 47.52 and 963.17 μmol/L. The average value was an impressive 371.82 ± 328.88 μmol/*L. prior* to treatment, the recorded cholesterol levels varied greatly, with the lowest at 4.9 mmol/L and the highest at 27.15 mmol/L. The average level was a staggering 13.36 ± 6.03 mmol/L, showing a wide range. The LDL-C levels were equally variable, ranging from 2.81 to 12.08 mmol/L, with an average of 5.98 ± 2.53 mmol/L. Among the 12 individuals examined, there was a wide range of Lp(a) levels, from 7.02 to 1020.00 mmol/L. Interestingly, two of these patients (16.7%) had extremely high Lp(a) levels exceeding 300 mmol/L. Among the 18 patients, only 2 (11.1%) had hemoglobin levels less than 110 g/L (< 6 years), and 2 others (11.1%) had hemoglobin levels ranging from 110 to 120 g/L (for ages 6 and over), indicating the presence of anemia. A substantial majority of 14 individuals (77.8%) had normal hemoglobin levels. Before treatment, we examined the platelet levels of 18 patients. Only one patient (5.6%) had a lower than expected platelet count of 90 × 10^9^/L, and the remaining 17 patients (94.4%) had a normal platelet count. The results for thyroid activity and liver function tests were within the optimal range.

Each patient received an abdominal ultrasound, as well as a cardiac and carotid artery ultrasound. In addition, fundus examinations were conducted on 14 patients. Two patients (7.7%) were diagnosed with aortic regurgitation, and the remaining 24 patients (92.3%) had no signs of abnormalities. Furthermore, there was thickening in the intima media of the bilateral carotid artery in 4 of the patients (15.4%), and in one patient (3.9%), there was thickening specifically in the right carotid artery. Notably, thickening of the carotid artery walls and plaque buildup in the unnamed right artery were observed in a 12-year-old patient (patient 3). None of the patients had splenomegaly or fundus vascular sclerosis.

### Genotypes

Among the 24 individuals (92.3%) who underwent genetic testing, 19 (79.2%) were found to have compound heterozygous variants, and 5 (20.8%) had homozygous variants. According to the genetic report, a single individual possessed a perplexing combination of compound heterozygous variants (c.1189C > T (p.Gln397Ter277), c.1240C > G (p.Leu414Val) and c.63 + 217C > T) in the *ABCG*8 gene, along with a single heterozygous variant (c.904 + 5G > C) in the *ABCG*5 gene. This patient was ultimately categorized as having an *ABCG*8 variant. Among all 24 patients, the majority (15, 62.5%) had *ABCG*5 variants, and the remaining patients (9, 37.5%) had *ABCG*8 variants. Interestingly, among these patients, the highest proportion (7, 29.2%) had both nonsense and missense variants, and the rest had a mix of other variant types, such as missense variants (6, 25.0%), nonsense variants (3, 12.5%), splicing and missense variants (3, 12.5%), splicing and nonsense variants (1, 4.2%), splicing, nonsense and missense variants (1, 4.2%), missense and frameshift variants (1, 4.2%), missense and synonymous variants (1, 4.2%) and splicing variants (1, 4.2%). This demonstrates the complex and diverse nature of the genetic variations present in this group of patients, showing the highest level of intricacy.

**Fig. 2:**
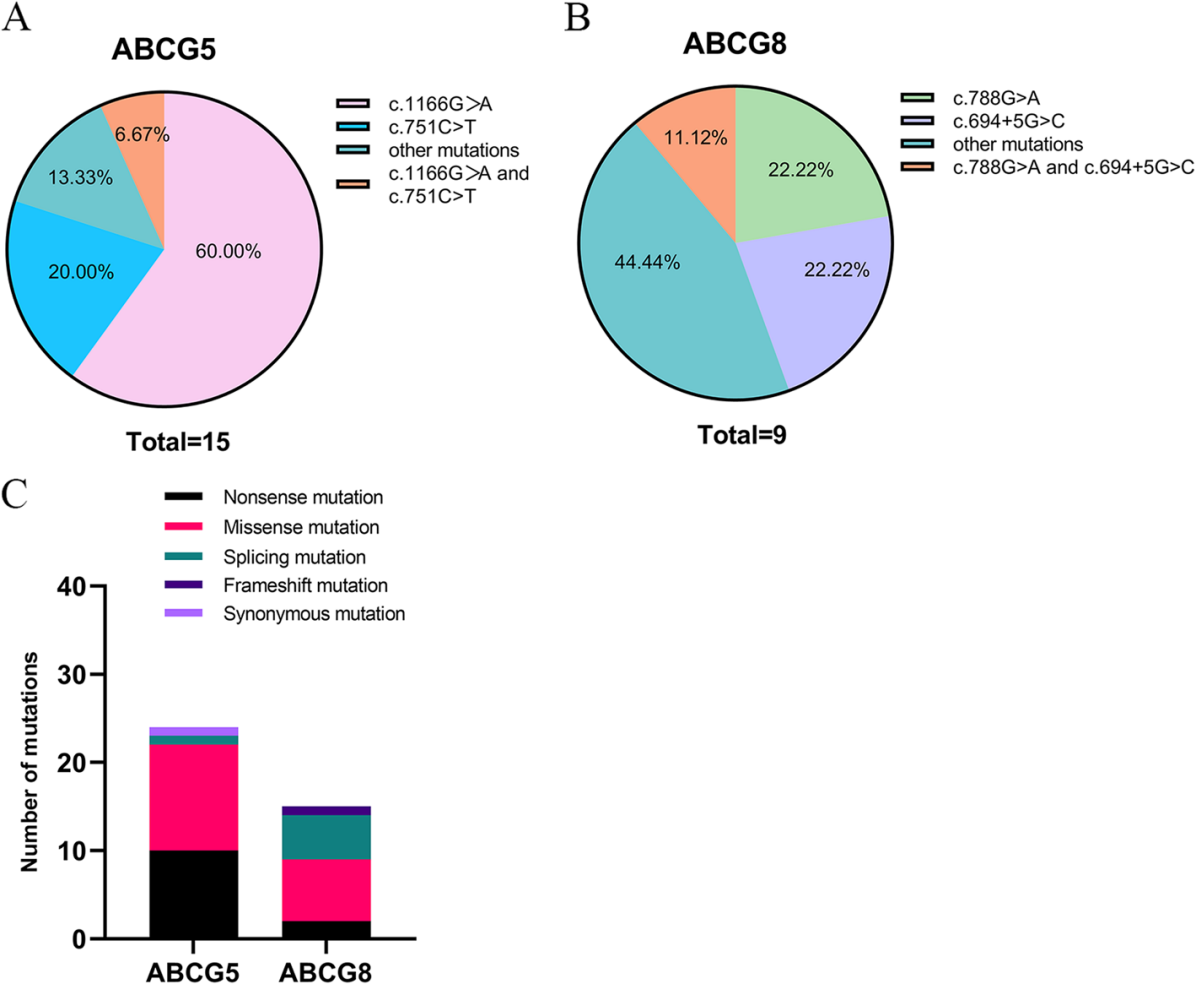
Variants in patients with sitosterolemia **A** Different types of variants in *ABCG*5. **B** Different types of variants in *ABCG*8. **C** Variant ratios of nonsense variants, missense variants, splicing variants, frameshift variants and synonymous variants in *ABCG*5 and *ABCG*8 patients

**Fig. 3:**
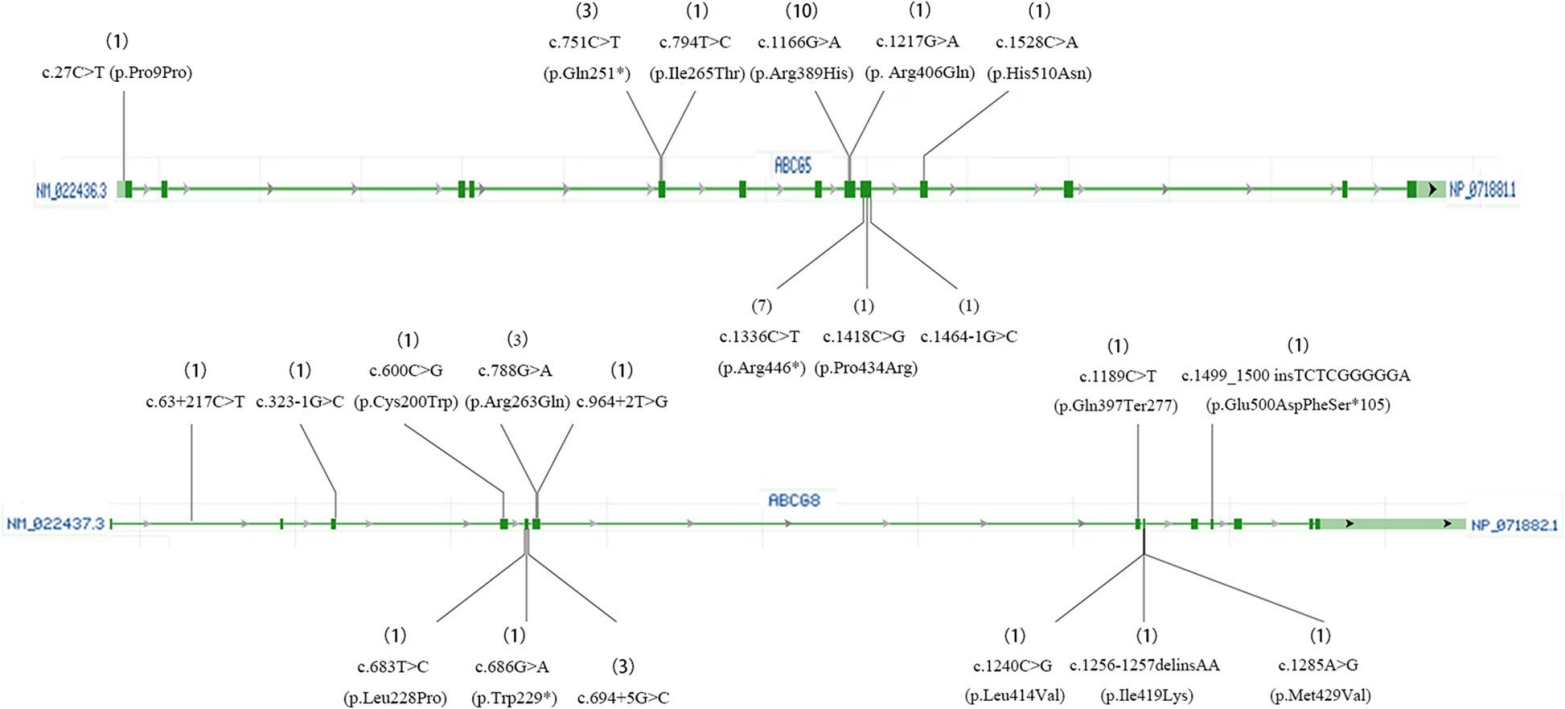
Different variant locations in the *ABCG*5 (A) and *ABCG*8 (B) genes

Among the 15 patients with type 2 sitosterolemia and *ABCG*5 variants, the variant c.1166G > A (p.Arg389His) was the most common and was present in 10 patients (66.7%). The second most common variant was c.1336C > T (p. Arg446*), which was present in 7 patients (46.7%), followed by c.751C > T (p. Gln251*), which was identified in 4 patients (26.7%) (Fig. 2A). Among the 8 patients who were diagnosed with type 1 sitosterolemia, c.788G > A (p.Arg263Gln) and c.694 + 5G > C were the most commonly observed *ABCG*8 variants, with a total of 3 (37.5%) patients (Fig. 2B). A visual representation of all variant types can be found in Fig. 3.

### Genotype–phenotype relationship

Among the 15 individuals who were diagnosed with type 2 sitosterolemia and who carried *ABCG*5 variants, a large majority (11, 73.3%) had developed xanthomas, and a significant number (5, 33.3%) were affected by arthritis. Additionally, a minority (3, 20.00%) experienced growth restriction, and one individual (6.7%) suffered from thrombocytopenia. Furthermore, numerous patients (3, 20%) reported experiencing anemia, and a small percentage (2, 13.3%) displayed high levels of Lp(a). In addition, 2 individuals (13.3%) exhibited cardiac ultrasound abnormalities, and 5 individuals (33.3%) had thickening of the bilateral carotid arteries. Among the 8 patients who carried *ABCG*8 variations, xanthomas were present in the majority (6, 66.7%) of individuals, and arthritis, growth restriction, and local thickening of the bilateral carotid arteries were found in 2 (22.2%) patients each. The research did not present any information regarding conditions such as low platelet counts, low red blood cell counts, abnormally high levels of Lp(a) (over 300 mg/L) or anomalous results from cardiac echocardiography. Further analysis, as shown in Table 2, did not indicate any significant differences in these traits among patients with *ABCG*5 and *ABCG*8 variations. We examined the differences in variant types between the *ABCG*5 and *ABCG*8 genes (Fig. 2C). Nonsense variants were more prevalent in *ABCG*5 (*P* = 0.09) than in *ABCG*8, and splicing variants were more frequently found in *ABCG*8 (*P* = 0.01). There was no significant difference in the frequency of missense variants, frameshift variants or synonymous variants (*P* > 0.05).

**Table 2:**
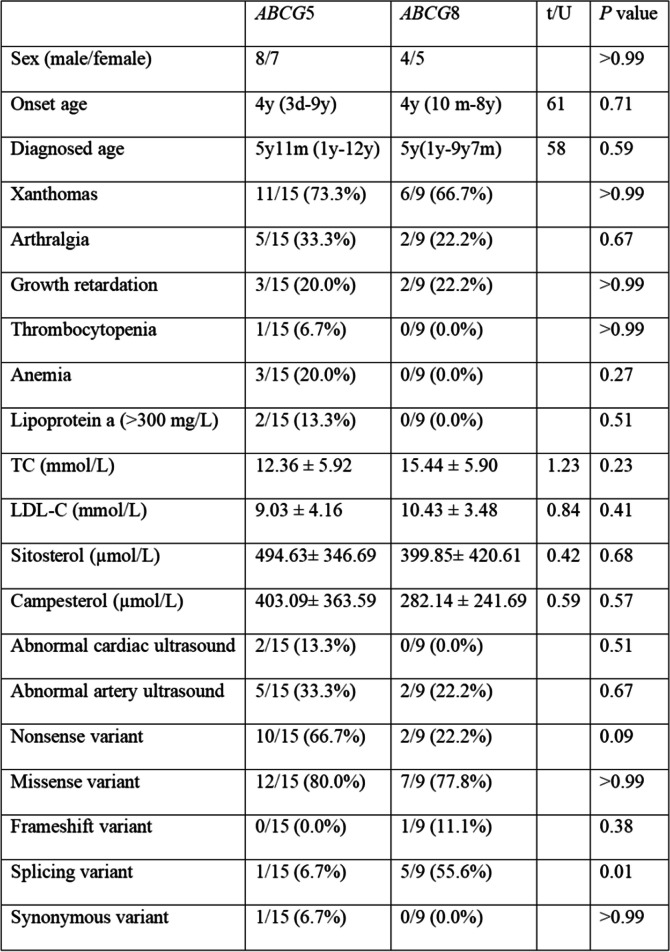
Clinical data of patients with *ABCG*5 and *ABCG*8 variants

Age at onset and age at diagnosis were analyzed using the Mann–Whitney test, and the corresponding U and *P* values were obtained. TC, LDL-C, sitosterol and campesterol levels were analyzed by unpaired t tests, and the corresponding t and *P* values were obtained. Other data were analyzed by exact FISH, and only *P* values were obtained

Table 3 shows a comparison between the clinical information of patients who had the most common harmful variation, c.1166G > A (p.Arg389His), and that of patients who had different variations. An astonishing 80.0% of patients with that variant exhibited xanthomas. Additionally, 40.0% presented with arthritis, and 30.0% presented with growth restriction. Additionally, 10.0% of patients experienced thrombocytopenia, 20.0% had high Lp(a) levels, another 20.0% exhibited abnormalities in their cardiac ultrasound results, and a striking 30.0% showed thickening of both carotid arteries. Among the patients included in the study with other variant, 60.0% had xanthomas, 20.0% had arthritis, 20.0% had anemia, and 40.0% had thickening of the bilateral carotid arteries. The occurrence of growth restriction, thrombocytopenia, high Lp(a) levels, and abnormalities in cardiac ultrasound results were not documented. It is intriguing to note that individuals with the variant c.1166G > A (p.Arg389His) exhibited a greater likelihood of having increased levels of Lp(a), as well as experiencing xanthomas, joint pain, stunted growth, and elevated levels of sitosterol and campesterol. The disparities, although observable, did not show statistical significance (*P* > 0.05). Among all patients, only 3 harbored with the common pathogenic variant c.751C > T (p.Gln251*), and a slightly greater number of 4 patients harbored the variant c.788G > A (p.Arg263Gln). As a result, a single statistical comparison was not conducted. The variant c.1336C > T (p. Arg446*) was frequently observed together with c.1166G > A (p. Arg389His); therefore, it was not subjected to statistical comparison with any other variants.

**Table 3:**
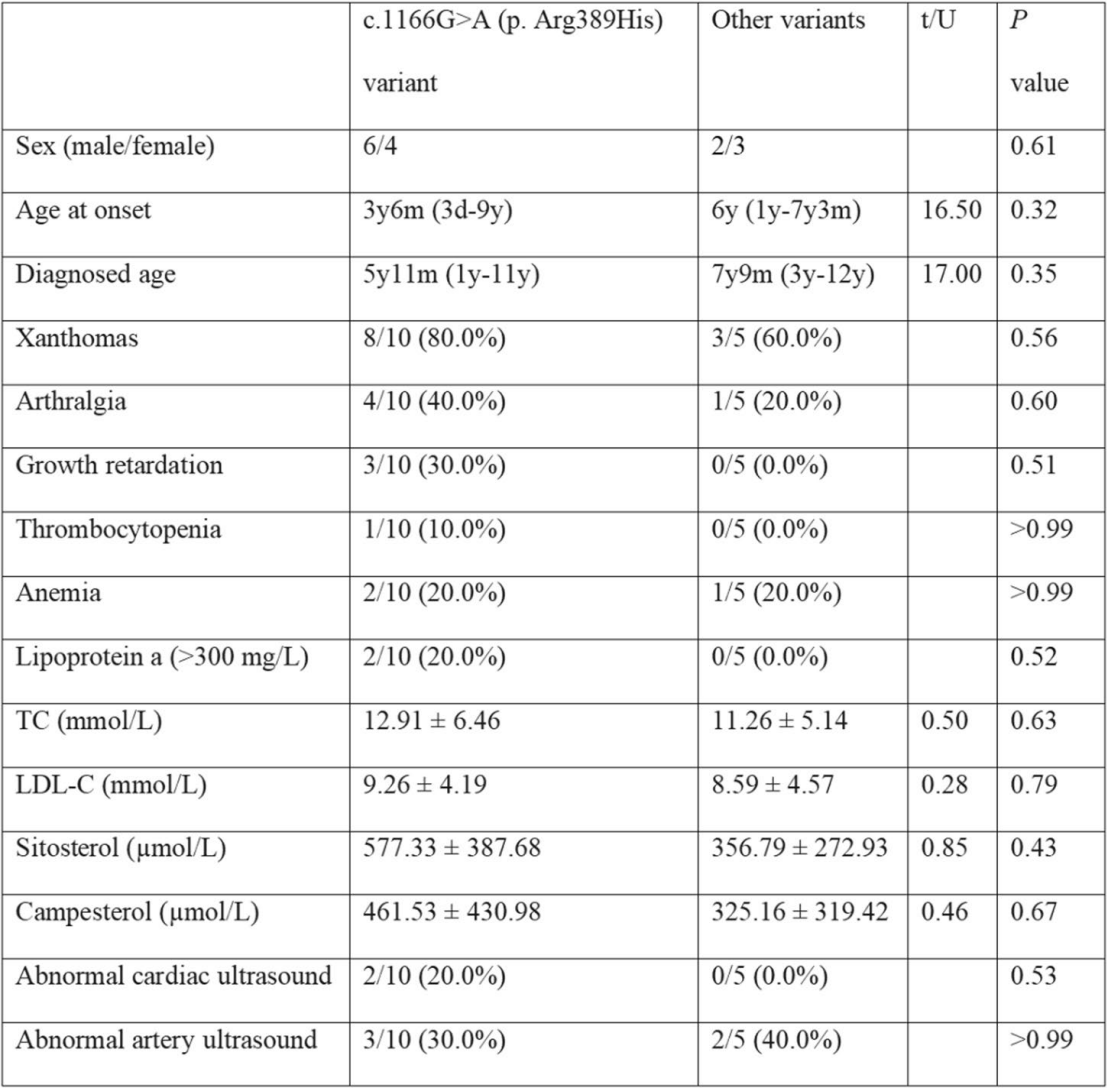
Clinical data of patients with c.1166G > A (p.Arg389His) and other variants of *ABCG*5

Age at onset and age at diagnosis were analyzed using the Mann–Whitney test, and the corresponding U and *P* values were obtained. TC, LDL-C, sitosterol and campesterol were tested by unpaired t tests, and the corresponding t and *P* values were obtained. Other data were analyzed by exact FISH, and only *P* values were obtained

### Treatment and follow-up

**Fig. 4:**
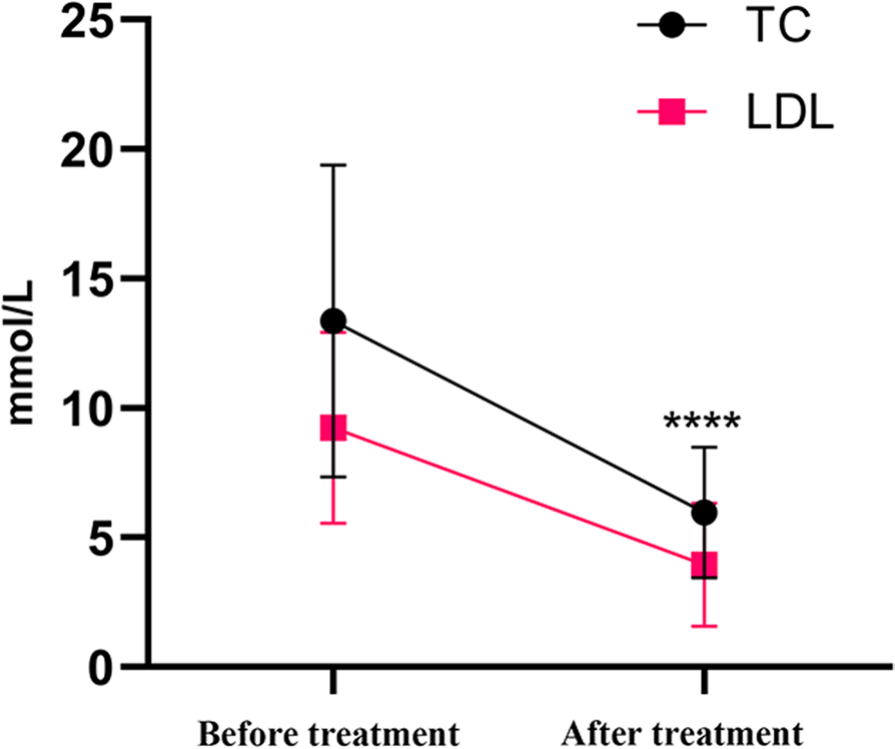
Total cholesterol (TC) and low-density lipoprotein cholesterol (LDL-C) levels were measured before and after treatment

Most patients (11, 42.3%) primarily received ezetimibe, either alone or in conjunction with other lipid-lowering medications, for treatment. Another group (13, 50.0%) focused solely on basic dietary management, and a select few used either simvastatin (1, 3.9%) or cholestyramine (1, 3.9%) alone. The levels of total cholesterol and LDL-C were assessed before and after treatment. For most patients, blood lipid levels can be controlled within 3 to 6 months. Following a change in diet or a combination of ezetimibe, the treatment led to a marked reduction in the levels of cholesterol and low-density lipoprotein compared to the levels seen before the treatment (*P* < 0.0001), as shown in Fig. 4.

## Discussion

To date, relatively few articles in China have systematically studied childhood sitosterolemia via genotype and phenotype analysis, and the results from clinical studies have strongly supported developments in the diagnosis and treatment of this disease in China. Compared to domestic data, the percentage of patients with sitosterolemia included in this study was high. The fact that almost 10% of the FH children at the Children’s Hospital of Zhejiang University School of Medicine had this condition raises the possibility that the prevalence of sitosterolemia is not fully recognized.

The diagnosis process varied from one to 12 years, with an average of 5.9 years. Young patients frequently present with physical signs of elevated lipid levels and the formation of yellow bumps called xanthomas during their initial diagnosis. This study revealed that xanthomas were generally the primary indicator, with an average onset age of 3 years. Sometimes, this situation is incorrectly identified as a skin ailment and is subsequently eradicated. Cerebrotendinous xanthomatosis is also a congenital lipid metabolism disorder, and this condition and xanthoma often occur together. However, patients with infantile diseases often exhibit symptoms of chronic diarrhea, and juvenile patients often exhibit clinical manifestations of cataracts. The initial symptoms are mainly unstable walking, cataracts or blurred vision, and decreased intelligence; however, none of our patients experienced these issues. Hematological sicknesses have been observed in patients who do not have xanthomas [17]. In this study, a single child who developed anemia without xanthomas was observed, highlighting the complexity of this disease. Among all the patients, a small percentage experienced joint pain and limitations in growth and development, indicating the variability of the progression of the illness. Current studies on the underlying mechanisms of joint pain and stunted growth due to sitosterolemia are limited. Patients sought treatment at the hospital after discovering high blood lipid levels during routine physical examinations. Patients with these characteristics tend to show clinical symptoms at an early age, usually before the age of 10, and are often mistakenly diagnosed with FH. It is quite intriguing that autosomal recessive diseases, unlike the predominantly autosomal dominant FH, which shows a distinct familial pattern, typically lack a genetic background (excluding elderly individuals with elevated blood lipid levels). Therefore, when identifying patients with xanthomas, joint pain, stunted growth, hyperlipidemia without a known family history, or high levels of lipids despite no inherited background, the possibility of sitosterolemia should be acknowledged.

While some may argue that the toxicity of sitosterol is not of great concern, the introduction of external sitosterol has been shown to have beneficial therapeutic effects on both arteries and endothelial cells [18, 19]. If left untreated, individuals with sitosterolemia have an increased chance of developing early-onset atherosclerosis compared to those without this condition. Currently, there is conflicting evidence on the impact of sitosterol, in addition to cholesterol, on atherosclerosis. Phytosterols can enter the lining of blood vessels, stimulate the production of foam cells, release inflammatory molecules that attract immune cells, and trigger the development of both atherosclerosis and xanthomas [20, 21]. While some studies have indicated that sitosterol treatment may result in the involvement of the aorta or valves [22, 23], this study observed only 2 patients with aortic regurgitation. Six individuals in the investigation had thickening of either their unilateral or bilateral carotid arteries. Interestingly, one patient exhibited plaque formation specifically in the right unnamed artery. This indicates a significantly elevated risk of atherosclerosis and highlights the urgency for prompt detection and intervention. Eberhard Windler et al. [24] studied whether phytosterols can increase the risk of atherosclerosis in the same manner as cholesterol can. However, this conclusion is still controversial. Thus far, whether phytosterols have long-term effects on the human body has not been elucidated. Therefore, it is unclear whether lowering blood phytosterol levels to within the normal range is necessary for pediatric treatment. At concentrations of 723–960 μmol/L, phytosterols have been found to potentially result in the formation of xanthomas. However, significantly elevated cholesterol levels, namely, 10.34 mmol/L or higher, are detrimental to the body because compared with cholesterol, sitosterol may have a stronger negative impact on human endothelial cells [25, 26]. Research indicates that individuals diagnosed with xanthoma exhibit elevated levels of inflammation, resulting in a 3.2-fold increase in their susceptibility to cardiovascular disease [27]. To date, there have been no documented cases of early-onset artery blockage in individuals with sitosterolemia who do not have visible xanthoma on the skin [10]. This research aligns with previous studies, as individuals with xanthomas were the only ones who exhibited cardiac ultrasound abnormalities and thickening in both carotid arteries. This could indicate a heightened risk for cardiovascular issues. Surprisingly, only a small number of patients sought medical treatment for joint pain or impaired growth. Regular analysis of blood lipids and phytosterols, along with frequent carotid and cardiac ultrasounds and fundus exams, are routine procedures.

Lp(a) has been identified as a distinct contributor to the development of cardiovascular issues and negative health outcomes [28, 29]. The research revealed that a mere 2 individuals had elevated lipoprotein levels (≥300 mg/L), an alarming discovery that warrants consistent, continuous monitoring to avoid potential cardiovascular complications. Additionally, it is of utmost importance to closely monitor the width of the central layer of the carotid artery. Any indication of thickening or plaque formation in this layer is a clear warning sign, requiring heightened vigilance.

Nonsense variants in *ABCG*5 were highly prevalent (*P* = 0.09, though not notably distinct), possibly because of the limited number of samples. However, instances of splicing variants were more commonly observed in *ABCG*8. The correlation between genotypes and phenotypes for *ABCG*5 and *ABCG*8 variations was analyzed in 24 individuals, and no noteworthy discrepancies were found. The common variant c.1166G > A (p.Arg389His) in *ABCG5* and other variants were also analyzed, and no discernible physical variations were observed across the various genotypes. This results of this study results must be confirmed because there are only a small number of individuals with rare diseases, causing a high level of complexity and unpredictability in the data.

The most effective treatments involve limiting the intake of plant sterols and cholesterol. Those with sitosterolemia should adhere to a strict diet and avoid phytosterol-heavy foods such as corn oil, peanuts, soybeans, and sesame oil, as well as cholesterol-rich foods such as animal liver, eggs, and even shellfish [30]. If cholesterol and sitosterol levels are not effectively managed, the combined use of ezetimibe is imperative. The prescribed dose for treatment, either 5 mg a day or 10 mg a day, results in a favorable response for the majority of individuals [31]. Unfortunately, it appears that younger patients, particularly those younger than 2, do not respond well to drug treatment [17, 32–34]. Most of our patients received a combination of ezetimibe therapy and stringent dietary management. This approach resulted in a notable decrease in both total cholesterol and LDL-C levels, demonstrating the importance of treating and tracking young patients. Nonetheless, a few patients continued to exhibit elevated levels of sitosterol despite undergoing both ezetimibe treatment and strict dietary control.

## Strengths and limitations

The strengths of the research are that medical centers have drawn in a diverse array of patients with varying forms of sitosterolemia from all corners of the nation, and a considerable number and variety of patients were included in this study. Moreover, we followed up and compared the clinical manifestations and treatment outcomes of the patients. Few complications and a delayed development of atherosclerosis benefited from early diagnosis, medication and diet control. Images and incidence rates of different positions of xanthomas are also presented in the article. Currently, there are few studies examining the differences in clinical effects between patients harboring *ABCG*5 and *ABCG*8 variants or between those harboring c.1166G > A (p.Arg389His) and other variants in *ABCG*5. We analyzed these comparisons and concluded that there was no difference. This has greatly promoted research on genotypes and phenotypes in China. There are several limitations to note in this research. First, some of the cases examined were based on past experience, and certain data points were not fully recorded. Moreover, there was a disparity among the patients in terms of their blood sitosterol levels, as there was no consistent measurement before and after treatment. Last, the sample size was not large enough to confidently determine any differences in how genotype-phenotype relationships are connected.

## Conclusion

In summary, sitosterolemia should be considered for individuals with xanthomas and high cholesterol levels. Phytosterol testing and genetic analysis are important for early detection. Differences in clinical features and laboratory and ultrasound indicators among patients with different variants were not found. Managing one’s diet and taking ezetimibe can well control blood lipids. Early diagnosis, dynamic monitoring of laboratory and ultrasound indicators, ezetimibe and diet control can significantly delay the occurrence of atherosclerosis and complications in patients with sitosterolemia.
